# Ghost Ileostomy Versus Protective Ileostomy in Rectal Cancer Followed by Low Anterior Resection: A Randomized Feasibility Trial

**DOI:** 10.1002/hsr2.71351

**Published:** 2025-10-13

**Authors:** Seyed Mostafa Meshkati Yazd, Mohammad Reza Keramati, Marzieh Ghanbari Ghalerudkhani, Reza Shahriarirad, Amir Parsa, Amir Keshvari

**Affiliations:** ^1^ Division of Colorectal Surgery, Department of Surgery Tehran University of Medical Sciences Tehran Iran; ^2^ Colorectal Research Center Tehran University of Medical Sciences Tehran Iran; ^3^ School of Medicine Shiraz University of Medical Sciences Shiraz Iran; ^4^ Thoracic and Vascular Surgery Research Center Shiraz University of Medical Sciences Shiraz Iran

**Keywords:** anastomotic failure, ghost ileostomy, protective ileostomy, rectal cancer

## Abstract

**Background:**

Anastomotic leakage remains a serious concern in rectal cancer patients undergoing neoadjuvant chemoradiotherapy (nCRT) followed by low anterior resection (LAR). To mitigate this risk, protective‌ ileostomy (PI) is often employed; ‌‌however, it carries its own complications and patient burden. This study aimed to evaluate the feasibility and clinical outcomes of ghost ileostomy (GI) as a potential alternative, aiming to reduce unnecessary stoma creation, related morbidity, readmission rates, and patient discomfort.

**Methods:**

In this randomized, non‐inferiority feasibility trial, patients with rectal cancer who had received nCRT and were scheduled for LAR were enrolled. Feasibility outcomes included recruitment rate, retention, intervention adherence, procedural fidelity, and safety. Safety outcomes compared GI and PI regarding anastomotic leakage and failure, bowel obstruction, acute tubular necrosis (ATN), and readmissions due to complications (primary endpoint).

**Results:**

Eighty patients were randomized from 87 eligible participants, with a recruitment rate of 5.71 patients per site‐month. GI was non‐inferior to PI regarding overall complications and readmissions (one‐tailed 90% CI; power > 80%) and also for obstruction and ATN (one‐tailed 95% CI; power > 80%). However, the PI group had a significantly shorter initial hospital stay than the GI group (*p* = 0.042).

**Conclusion:**

This trial supports the feasibility of a definitive future study. GI may serve as a safe and effective alternative to PI in selected low‐risk rectal cancer patients undergoing nCRT and LAR. Larger, multicenter trials are needed to validate these findings and further explore the clinical utility of GI.

## 1 Introduction

Colorectal cancer is the second leading cause of cancer‐related death in men and the third in women in the United States. Among the approximately 149,500 new cases diagnosed annually, rectal cancer accounts for nearly one‐third [1, 2].

Neoadjuvant chemoradiotherapy (nCRT) has become the standard of care for locally advanced rectal cancer, particularly in resectable stage II and III disease. The benefits of nCRT include increased rates of tumor resection and sphincter preservation, tumor downstaging, and reduced local recurrence, offering advantages over neoadjuvant chemotherapy alone [3]. Protective ileostomy (PI) is commonly recommended for these patients to mitigate the consequences of anastomotic leakage [4]; however, PI is associated with considerable risks. Nearly 50% of patients receiving a PI—many of whom have colorectal malignancy—experience complications such as intestinal obstruction, diarrhea, surgical site infection, electrolyte imbalance, and enterocutaneous fistula [5, 6].

Despite advances in surgical techniques and equipment, anastomotic leakage remains a frequent and serious complication following anterior resection, with reported incidence rates ranging from 1.6% to 20.5% [7, 8, 9]. Anastomotic leaks are linked to reduced survival and increased local recurrence due to extraluminal implantation of cancer cells in this potentially curative malignancy [10, 11]. In addition, readmission may be needed in patients undergoing ileostomy to resolve problems due to ileostomies failures, such as necrosis, prolapse, or intestinal obstruction in one‐sixth of cases. This causes many issues after surgery, leading to depression, social and even sexual problems that reduce their quality of life [11, 12]. Reoperation for ileostomy closure and following possible complications such as gastrointestinal obstruction and anastomosis leak should be considered.

Ghost ileostomy (GI), a pre‐stage ileostomy that can be activated if clinically indicated, offers a more flexible alternative by potentially reducing unnecessary ileal diversion [8]. To our knowledge, our study is among the limited randomized trials comparing PI and GI in rectal cancer patients undergoing nCRT and low anterior resection (LAR). Given ongoing debate about the routine use of temporary protective stomas, this pilot study was designed to evaluate the feasibility of larger definitive trials. It investigates whether GI can safely reduce the need for PI in patients at low risk for anastomotic leak, particularly those treated with nCRT, thereby decreasing ostomy‐related complications and patient morbidity. In addition to clinical outcomes, feasibility endpoints such as recruitment and retention rates, intervention adherence and fidelity, and safety were assessed. This study aims to assess the feasibility and safety of GI versus PI to potentially reduce unnecessary ostomy creation, associated complications, readmissions, and patient discomfort.

## 2 Methods

### 2.1 Trial Design

A feasibility trial was conducted according to the extention of Consolidated Standards of Reporting Trials (CONSORT) statement for randomized pilots and feasibility trials. This pilot trial had non‐inferiority and parallel design with allocation ratio one. This clinical trial was registered at the Iranian Registry of Clinical Trials on 10/01/2021. (Trial code: IRCT20120129008861N3).

### 2.2 Participants

In this prospective, randomized clinical trial, 80 patients with previous diagnoses of rectal cancer candidates for low anterior resection surgery in the colorectal division of the surgery departments of Imam Khomeini Hospital were evaluated by convenience sampling. Colorectal Surgery Department of Imam performed about 450 colon and rectal cancer surgery during 2024, and around 1900 colorectal cancer patients were managed during 2000–2024 in our department.

We designed this project as a pilot study based on the limited number of patients willing to participate in our study. The inclusion criteria consisted of rectal cancer patients who have undergone neoadjuvant chemoradiotherapy, and the anastomosis location should be between 5 cm or more from the anal verge. Patients were excluded from the study if surgery may be more risky than usual due to adhesions to adjacent organs or the spread of a tumor or abnormal bleeding (more than one litre), challenging pelvic dissection, excessive traction, and suspected anastomotic blood flow, or the presence of a hematoma. Patients were also excluded in cases of diabetes, body mass index (BMI) of above 35, age of above 75 years, American Society of Anesthesiology score of above 3, signs of obstruction or peritonitis, immunosuppressive diseases, and if the air leak test was positive, or the doughnut was incomplete at the end of the operation, emergencies or intestinal obstruction during operation.

### 2.3 Intervention

**Figure 1:**
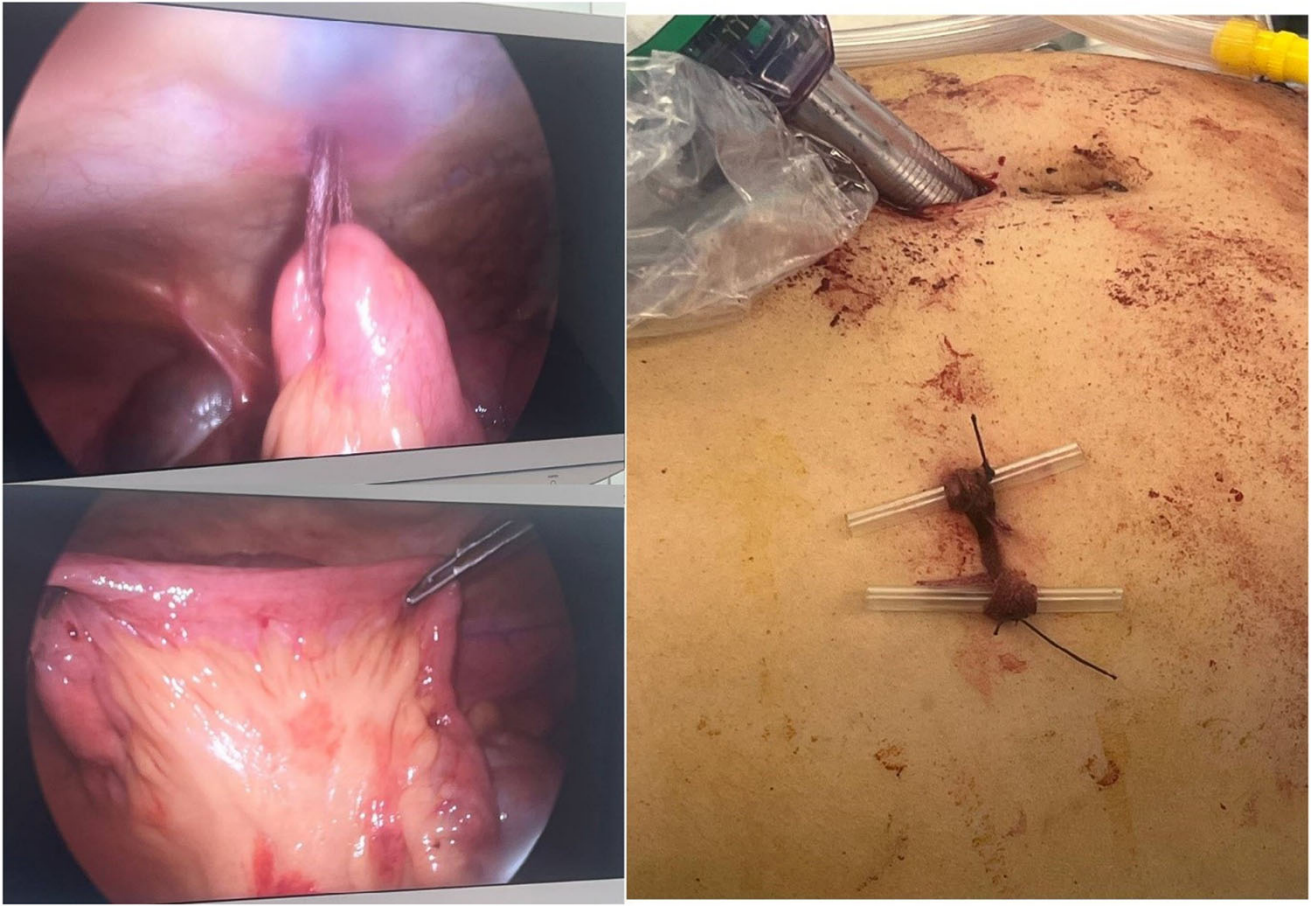
Minimal invasive Ghost ileostomy placement in patients with rectal cancer. The first step includes identifying the proper ileal loop and making a small hole in the mesentery. Thereafter, the rubber band through the small hole in the mesentery; Bringing out the two limbs of the rubber band through the abdominal wall, and then secured to the skin on a rod.

For GI insertion, after completing colorectal anastomosis, an intestinal loop approximately 30 cm from the ileocecal valve was identified, and a rubber band was gently passed around the terminal ileum through a window adjacent to the ileal wall without causing iatrogenic trauma to the mesenteric vessels. The rubber band was then exteriorized through a small hole in the right iliac fossa, secured to the skin on a rod, and removed after ten days. This procedure is easily performed during laparoscopy, where the terminal ileal loop is encircled with a rubber band and externalized through the trocar incision. If needed, the ileal loop can be externalized under local or general anesthesia to create a true loop ileostomy [8]. (Figure 1) The anastomosis height and the distance of the tumor from the anal verge were assessed by a colorectal surgeon using rectoscopy with flexible sigmoidoscopy. All the patients underwent standard resection on TME (total mesorectal excision) plan.

### 2.4 Outcomes

The outcomes of this pilot trial consisted of feasibility outcomes and clinical outcomes. The feasibility outcomes were as the following:

**Reqruitment rate:** The number of randomized cases *per* number of sites *per* months of enrollment

**Retention rate:** The proportion of the cases completed the protocol of the study

**Intervention adherence:** Not applicable as the intervention was surgical

**Intervention fidelity:** Not applicable as the intervention was surgical

**Safety:** Same with the cilinical outcomes including non‐inferiority regarding complications. The primary endpoint among them was readmission rate.

The results were collected and compared in the two groups in terms of the incidence of the anastomotic leak based on clinical symptoms (such as ileus, fever, tachycardia, peritonitis symptoms, gastrointestinal or purulent leakage from the drain), laboratory symptoms (increased C reactive protein (CRP), Erythrocyte sedimentation rate (ESR), and leukocytosis) and also imaging findings. The mean duration of surgery, the need for reoperation, the need for readmission due to complications of the operation (due to anastomosis or ostomy complications in short‐term follow‐up within 2 months), and different complications occurring in the two groups were compared.

All patients were visited at the colorectal clinic one and 2 weeks after discharge, and then 2 months after surgery. In post‐surgery visits, patients were evaluated in terms of clinical symptoms, laboratory symptoms and physical examination. In addition, all patients underwent flexible rectosigmoidoscopy in 8–10 weeks after surgery to evaluate the anastomosis site. Ultimently, all patients underwent standard surveillance visits like all rectal cancer patients [13].

### 2.5 Data Analysis

As a pilot feasibility study, the sample size of 80 patients was pragmatically determined based on the researchers′ estimation of recruitment capacity within a 1‐year single‐center sampling period. No a priori power calculation was performed, consistent with the exploratory nature of feasibility trials. Following completion of the study, a post hoc power analysis was conducted with a non‐inferiority approach [14, 15] sing the observed effect sizes, a non‐inferiority margin of 10% for proportional outcomes, 80% power, and one‐tailed 90% and 95% confidence intervals (CI). This analysis was not intended to validate the study results but rather to provide an estimate of the required sample size for future definitive trials.

Although blinding of surgeons and patients was not feasible due to the nature of the intervention, outcome assessors and data analysts were blinded to group allocation to reduce bias.

Data analysis was performed using SPSS software version 27. The statistical tests used to compare the results of the two groups included Chi‐square, Fisher′s exact test, independent t‐test, and logistic regression. A *P*‐value of less than 0.05 was considered statistically significant. While interpretation of confidence intervals is the recommended approach for clinical trials, the post hoc analysis was included here to contextualize the feasibility of detecting non‐inferiority in future studies, acknowledging its inherent limitations.

### 2.6 Ethical Considerations

In the study, the patient′s information was recorded confidentially, and there was no charge for patients in none of the stages of the study. Besides, written informed consent was obtained from all patients (or their legal representatives). The study was approved by the ethics committee of the Tehran University of Medical Sciences (IR. TUMS. IKHC. REC.1398.020) and conducted in compliance with the Declaration of Helsinki [16].

## 3 Results

**Figure 2:**
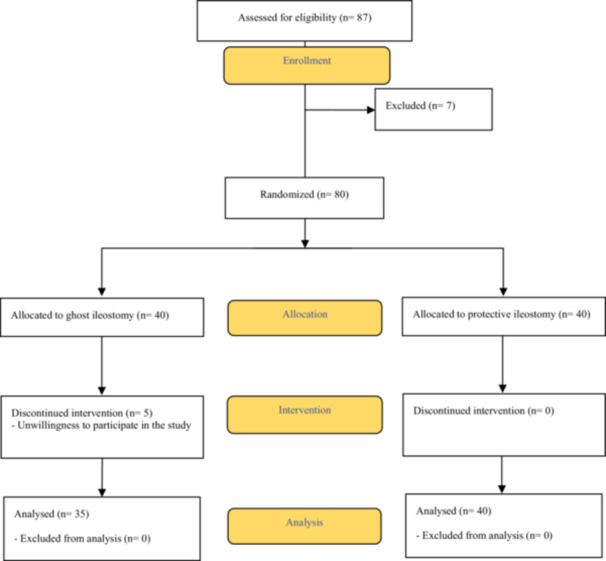
Consort Diagram of rectal cancer patients undergoing ileostomy after anastomosis.

A total of 80 patients diagnosed with rectal cancer met the inclusion criteria and were enrolled in the study. Patients were randomized to undergo either a protective ileostomy (PI) or a ghost ileostomy (GI). Among the 40 patients allocated to the GI group, five withdrew consent and were excluded from the final analysis. Therefore, 35 patients underwent GI following the creation and confirmation of a secure colorectal anastomosis. Figure 2 provides a flow diagram of the study population and allocation.

The feasibility outcomes of the study included recruitment and retention rates. The recruitment rate, based on the enrollment of 80 cases at a single center over a 14‐month period, was calculated as 5.71 patients per site‐month. The overall retention rate was 93.7%, with full retention in the PI group (100%) and a slightly lower rate of 87.5% in the GI group.

Patients ranged in age from 29 to 75 years, and their body mass index (BMI) varied between 16.9 and 34.0 kg/m². Table 1 presents a comparative overview of the demographic and clinical characteristics between the GI and PI groups. There were no statistically significant differences in baseline variables. However, the duration of hospitalization was significantly shorter in the PI group compared to the GI group, with a *p*‐value of 0.042.

**Table 1:** Demographic and clinical features of rectal cancer patients undergoing neoadjuvant chemoradiotherapy based on ghost ileostomy (GI) versus protective ileostomy (PI).

Variable		Total	Ileostomy; *N* = 75		*p* value*
			Ghost; *n* = 35	Protective; *n* = 40	
Age (years); mean ± SD		53.20 ± 12.43	53.37 ± 12.67	53.05 ± 12.37	0.912
Gender; *n* (%)	Male	52 (69.3)	23 (65.7)	29 (72.5)	0.525
	Female	23 (30.7)	12 (34.3)	11 (27.5)	
BMI (kg/m^2^); mean ± SD		24.74 ± 2.91	24.30 ± 2.09	25.13 ± 3.46	0.208
Operation duration (minutes); median [IQR]		180 [90]	184 [100]	180 [85]	0.594
Distance of tumor from the anal verge (cm); median [IQR]		9 [3]	9 [2.0]	9 [3.75]	0.652
Operation till discharge (days); median [IQR]		7 [3]	8 [2]	6 [3]	**0.042**
Surgery; n (%)	Laparoscopy	15 (20.0)	10 (28.6)	5 (12.5)	0.083
	Open	60 (80.0)	25 (71.4)	35 (87.5)	
Complication; n (%)	Total	10 (13.3)	3 (8.6)	7 (17.5)**	0.321
	Anastomosis failure	6 (8.0)	3 (8.6)	3 (7.5)	1.000
	Obstruction	4 (5.3)	0 (0)	4 (10.0)	0.118
	ATN	4 (6.3)	0 (0)	4 (10.0)	0.118
Readmission due to complication; n(%)		9 (12.0)	3 (8.6)	6 (15.0)	0.489

Abbreviations: ATN, Acute tubular necrosis; BMI, Body Mass Index; IQR, Interquartile range; SD, standard deviation.

* Chi‐square/Fisher′s exact test, or Mann‐Whitney/independent sample t‐test.

** Two patients with ATN complications also had anastomosis failure and obstruction complications.

In patients who underwent GI, the rubber band securing the externalized ileal loop was typically cut between 10 and 17 days postoperatively (mean 13.47 ± 2.23 days), depending on the results of the proctoscopic evaluation of the anastomosis and the patient′s overall clinical condition.

During the follow‐up period, three patients in the GI group experienced anastomotic failure. These events occurred at 14, 60, and 180 days postoperatively. None of these patients exhibited clinical signs such as abdominal distension, sepsis, or ileus during the initial postoperative hospitalization. The first patient presented with dysuria and pelvic discomfort approximately 2 weeks after surgery. Proctoscopic examination revealed a minor anastomotic disruption, and the patient was subsequently taken to the operating room for conversion to a PI. The patient was discharged 2 days later and underwent conservative management, including repeated proctoscopic evaluations and sponge insertion as (previously described by the authors [17], which resulted in progressive granulation tissue formation and eventual healing of the defect. The other two cases were diagnosed based on delayed symptoms. One patient reported persistent pelvic pain for 2 months, while the other experienced rectorrhagia and foul‐smelling anal discharge 6 months postoperatively. Both were readmitted, underwent diversion ileostomy, and received irrigation and sponge therapy during periodic proctoscopies until the anastomotic disruptions were resolved. All three patients eventually demonstrated complete anastomotic integration on follow‐up evaluations.

In the PI group, seven patients (17.5%) experienced complications. These included one case of anastomotic failure (2.5%), one case of bowel obstruction following ileostomy closure (2.5%), and one case of parastomal hernia accompanied by obstruction (2.5%). Four cases (10%) involved ATN, among which two were associated with anastomotic failure and obstruction—one occurring before and the other after ileostomy closure. Comparison of postoperative complications between the GI and PI groups revealed no statistically significant difference, with an odds ratio of 0.441, a 95% confidence interval of 0.105 to 1.860, and a *p*‐value of 0.256.

Given the pilot nature of the study, a post hoc power analysis was conducted based on the primary outcome of readmission rate, which was 8.6% in the GI group compared to 15% in the PI group. This analysis indicated that a sample size of 96 patients would be required to achieve 80% power at a one‐tailed 95% confidence interval. However, with a one‐tailed 90% confidence interval, a sample size of 70 patients would be sufficient. These findings support the non‐inferiority of the GI approach at the 90% confidence level. The results of power analyses for additional outcomes are summarized in Table 2, and detailed findings are provided in Supplement 1.

**Table 2:** Power analysis (total sample size needed) for detection of non‐inferiority on the basis of observered effect sizes, 80% power and 10% non‐inferiority limit.

Variable n(%)	Ileostomy; *N* = 75		Power analysis (total sample size needed), *n*	
	Ghost; *n* = 35	Protective; *n* = 40	One tailed 95% CI	One tailed 90% CI
Total complications	3 (8.6)	7 (17.5)	78	58*
Anastomosis failure	3 (8.6)	3 (7.5)	232	170
Obstruction	0 (0)	4 (10.0)	28**	22*
ATN	0 (0)	4 (10.0)	28**	22*
Readmission due to complication	3 (8.6)	6 (15.0)	96	70*

* Non‐inferior at one‐tailed 90% CI (calculated sample size lower than the pilot sample size),

** non‐inferior at one‐tailed 95% CI (calculated sample size lower than the pilot sample size)

## 4 Discussion

Given the importance of the topic, conducting a clinical trial was essential. However, due to the relatively low incidence of rectal cancer, initiating a full‐scale trial was not feasible. Therefore, a feasibility study was conducted to assess both the safety of the intervention (i.e., non‐inferiority in preventing complications) and the potential for adequate patient recruitment for future clinical trials. The observed recruitment rate of 5.71 patients per site‐month indicates that, in high‐volume rectal cancer centers, it would be possible to enroll approximately 137 patients per year using two sites. This recruitment potential is directly tied to the substantial annual caseload at our institution, which performs around 450 colorectal cancer surgeries annually. Consequently, the feasibility and scalability of future trials will largely depend on the patient volume of participating centers.

To the best of our knowledge, our study is among the first trials comparing PI and GI in rectal cancer patients undergoing neoadjuvant chemoradiotherapy (nCRT) followed by LAR. Another recent randomized pilot trial compared GI with conventional loop ileostomy in rectal cancer patients undergoing LAR/TME {Hüttner, 2024 #38}. Although their study was stopped early due to slow recruitment, outcomes showed no significant differences between groups regarding anastomotic leakage, postoperative morbidity, function, or quality of life. About 40% of GIs required conversion to a conventional stoma, but no terminal stomas were needed. Their findings suggest that ghost ileostomy is a safe and feasible alternative for selective ileostomy creation, though optimal patient selection remains a challenge{Hüttner, 2024 #38}. Given the ongoing debate surrounding routine PI creation in all patients, our study aimed to evaluate whether GI could serve as a viable alternative in low‐risk patients—particularly those treated with nCRT—by reducing unnecessary ostomy formation and its associated complications. One notable advantage of GI observed in our trial was a lower readmission rate, despite no significant difference in overall postoperative complications. However, this benefit came at the cost of a slightly longer initial hospitalization. Importantly, GI can help avoid the additional hospitalization required for PI reversal. While PI does not reduce the incidence of anastomotic leakage, it may mitigate its consequences. GI, in contrast, appears to be a favorable alternative in carefully selected patients, as it minimizes the need for multiple surgeries and hospital admissions. All PI patients in our study required at least one readmission for stoma reversal. Furthermore, GI is quicker and technically simpler to perform than PI and can be easily converted to a functioning stoma under local anesthesia if leakage is suspected. Although neither approach alters the leakage rate, using GI may prevent unnecessary laparotomies.

Constructing a GI did not reduce complication‐related readmissions compared to PI. However, only three patients (8.6%) in the GI group required readmission, all due to anastomosis‐related issues, and no ileostomy‐related complications led to readmission. In contrast, all patients in the PI group were readmitted at least once for stoma reversal.

A meta‐analysis by Pisarska et al. reported no significant difference in overall readmission rates between GI and PI groups [18]. In our study, although the initial postoperative hospitalization was longer in the GI group—likely due to closer monitoring—the total length of hospital stay was ultimately longer in the PI group, primarily due to the need for readmission for stoma reversal. Notably, some GI patients required conversion to PI and brief rehospitalization (approximately 2 days) for the management of anastomotic leakage. While these cases were included in the analysis, they may introduce potential bias. The mean operative time was shorter in the GI group, though the difference did not reach statistical significance. In line with previous studies, the overall treatment duration tended to be shorter in patients managed with GI [19].

There was no significant difference in the overall complication rates between the GI and PI groups. However, the most clinically relevant complication observed was gastrointestinal obstruction in PI patients, which led to hospital readmissions and associated morbidities, including ATN. A 2021 meta‐analysis also indicated that while diverting ileostomy may marginally reduce the anastomotic leak rate, it introduces a range of additional complications that may offset its protective benefits [20].

The effect of implanting GI in reducing complications is debatable. In some studies, there is a higher morbidities rate in PI than in GI [18]. According to Mari et al. [8], there was no significant difference in the amount of leakage in the cause of GI implantation versus non‐implantation. At the same time, it has been demonstrated that in cases of anastomotic leakage, patients with GI experienced less severe leakage and lower rates of peritonitis compared to those with PI. Additionally, the need for re‐laparotomy was effectively eliminated in the GI group. According to Gullà et al. [19], ostomy‐related complications in PI patients were 37% compared to 5.5% in GI patients. Overall morbidity was 40.7% in PI versus 5.5% in GI. The overall duration of treatment was more remarkable in PI, and the quality of life was significantly better in GI than in PI. We did not notice any significant difference in the overall complication rate between GI and PI.

Most anastomotic leak complications occurred long after the initial surgery, suggesting that ileostomy construction may not effectively prevent delayed anastomotic failure. Notably, in one of the three patients with complications, the diagnosis of anastomotic leakage was made 180 days postoperatively—well beyond the typical ileostomy reversal period. In this case, even if a protective ileostomy had been constructed, it likely would have been reversed by the time the complication developed, offering no preventive benefit.

This study primarily focused on the readmission rate and major postsurgical complications. While no statistically significant difference was observed between the GI and PI groups in terms of complications and complication‐related readmissions, the overall readmission rate was lower in the GI group. Importantly, all PI patients required rehospitalization for stoma reversal, which inherently contributed to a higher readmission rate. Although our findings suggest a potential advantage of GI in reducing hospital readmissions, further investigation is needed to assess its effectiveness in preventing long‐term complications such as delayed anastomotic failure or fistula formation.

As a pilot trial, the most notable limitation was its limited statistical power to detect a significant benefit of ghost ileostomy, particularly concerning anastomotic leaks. However, the sample size was not determined by an a priori calculation, and the post hoc power analysis performed was exploratory, serving only to inform the design of future definitive trials rather than to validate the present findings Given the limited number of rectal cancer patients eligible and consenting to this novel intervention, the sample size constraint was unavoidable and somewhat restricts the generalizability and statistical significance of the findings. Nevertheless, the current sample size was appropriate for assessing feasibility [21, 22]. Furthermore, all patients excluded after randomization withdrew due to personal unwillingness to continue participation, and none were excluded during or after the initiation of GI insertion. Given the clinical importance of the topic, conducting this pilot study was essential to provide preliminary data. It is also important to note that our evaluation specifically targeted patients considered at lower risk for anastomotic leakage, which explains the application of strict and detailed exclusion criteria. Collaborative studies across different countries and pooled data analyses can help overcome the current limitations. Additionally, future research should explore other influential factors in surgical outcomes, such as the type of anesthesia and specific operative techniques.

## 5 Conclusion

In summary, the PI group showed no significant inferiority compared to the GI group, with both groups exhibiting similar rates of postoperative complications. However, all PI patients required rehospitalization for ileostomy reversal, whereas no such hospitalization was needed in the GI group—suggesting potential advantages for GI in terms of cost reduction and quality of life. While the role of GI in reducing ostomy‐related and other complications remains debated, our findings support its use as an alternative approach in selected rectal cancer patients undergoing low anterior resection, particularly low‐risk cases receiving nCRT. Moreover, based on feasibility outcomes, conducting a definitive trial appears practical and justified.

## Author Contributions

**Seyed Mostafa Meshkati Yazd:** conceptualization, data curation. **Mohammad Reza Keramati:** conceptualization, validation. **Marzieh Ghanbari Ghalerudkhani:** investigation. **Reza Shahriarirad:** writing – original draft, writing – review and editing, supervision. **Amir Parsa:** writing – original draft. **Amir Keshvari:** supervision, conceptualization.

## Ethics Statement

In the study, the patients′ information was recorded confidentially, and there was no charge for patients in none of the stages of the study. Besides, written informed consent was obtained from all patients (or their legal representatives). The study was approved by the ethics committee of the Tehran University of Medical Sciences (IR. TUMS. IKHC. REC.1398.020) and conducted in compliance with the Declaration of Helsinki [16]. This study was also enlisted in the registry for clinical trial (Code: IRCT20120129008861N3).

## Consent

The authors have nothing to report.

## Conflicts of Interest

The authors declare no conflicts of interest.

## Transparency Statement

The lead author Amir Keshvari affirms that this manuscript is an honest, accurate, and transparent account of the study being reported; that no important aspects of the study have been omitted; and that any discrepancies from the study as planned (and, if relevant, registered) have been explained.

## Supporting information

supmat.

## Data Availability

The datasets used and/or analyzed during the current study are available from the corresponding author on reasonable request and with permission from the Research Ethics Committee of the School of Medicine‐Tehran University of Medical Sciences.
