# Supplementary material for: Ghost Ileostomy Versus Protective Ileostomy in Rectal Cancer Followed by Low Anterior Resection: A Randomized Feasibility Trial
Source: Health Sci Rep. 2025 Oct 13;8(10):e71351. doi: 10.1002/hsr2.71351 (PMC12516217; doi:10.1002/hsr2.71351)
Supplement: Supplementary file 1 — supmat. [file HSR2-8-e71351-s001.docx]

Supplementary materials

Supplement 1. Power analysis results using sealed envelop website available from: https://www.sealedenvelope.com/power/binary-noninferior/
